# Fournier’s gangrene at a tertiary health facility in northwestern Tanzania: a single centre experiences with 84 patients

**DOI:** 10.1186/s13104-015-1493-1

**Published:** 2015-09-28

**Authors:** Phillipo L. Chalya, John Z. Igenge, Joseph B. Mabula, Samson Simbila

**Affiliations:** Department of Surgery, Catholic University of Health and Allied Sciences-Bugando, Mwanza, Tanzania; Department of Urology, Bugando Medical Centre, Mwanza, Tanzania

**Keywords:** Fournier’s gangrene, Predisposing factors, Management, Prognostic factors, Tanzania

## Abstract

**Background:**

Fournier’s gangrene (FG) is a rare, rapidly progressive, necrotizing fasciitis of the external genitalia and perineum, leading to soft-tissue necrosis. Despite antibiotics and aggressive debridement, the mortality rate of FG remains high. This study describes our experiences in the management of FG and identifies prognostic factors.

**Methods:**

This was a descriptive retrospective study of patients with FG treated at Bugando Medical Centre between November 2006 and April 2014.

**Results:**

A total of 84 patients (M:F = 41:1) were studied. The median age was 34 years (range 15–76 years). The most common predisposing factor was diabetes mellitus (16.7 %). Nine (11.3 %) patients were HIV positive. Bacterial culture results were obtained in only 46 (54.8 %) patients. Of these, 38(82.6 %) had polymicrobial bacterial growth while 8 (17.4 %) had monomicrobial bacterial growth. Escherichia coli (28.3 %) were the most frequent bacterial organism isolated. All the microorganisms isolated showed high resistance to commonly used antibiotics except for Meropenem and imipenem, which were 100 % sensitive each respectively. All patients were treated with a common approach of resuscitation, broad-spectrum antibiotics, and wide surgical excision. The median length of hospital stay (LOS) was 28 days and mortality rate was 28.6 %. Systemic inflammatory response syndrome and diabetes mellitus were significantly associated with prolonged LOS (p < 0.001), whereas advancing age (>60 years), late presentation (>48 h), systemic inflammatory response syndrome on admission, diabetes mellitus, extension of infection to the abdominal wall, FG severity score >9 and HIV infection with CD4 count <200 μl/cells) were independent predictors of mortality (p < 0.001).

**Conclusion:**

Fournier’s gangrene remains a very severe disease with high mortality rates. Early recognition of infection associated with invasive and aggressive treatment is essential for attempting to reduce mortality rates associated with this disease in our setting.

## Background

Fournier’s gangrene is a rare and often fulminant necrotizing fasciitis of the perineum, perianal and genital regions, which may extend up to the abdominal wall between the fascial planes [1]. The condition was first described as a disease of young adults of unknown cause by Fournier in 1888 [2]. It is secondary to polymicrobial infection by aerobic and anaerobic bacteria with a synergistic action [3–6]. Fournier’s gangrene commonly affects men but does not spare women who constitute about 10 % and also afflicts children. It occurs commonly among those in their 5th and 6th decade [7].

The cause of infection is identifiable in 95 % of cases, mainly arising from anorectal, genito-urinary and cutaneous sources [8]. Predisposing factors include diabetes mellitus, local trauma, urine leakage, perirectal or perineal surgery, ex-tension of periurethral, anal infection, anorectal abscess, genitourinary infection, alcoholism, immunosuppression and renal or hepatic disease [4, 7, 9, 10]. Diagnosis is based on clinical signs and physical examination. Radiological methods may help to delineate the extent of the disease but false negatives may happen. Several reports tried to evaluate the usefulness of diverse scoring systems. Fournier’s Gangrene Severity Index (FGSI) has become a standard for researchers, being routinely published in FG literature and is considered as a good predicting tool [11–14].

Despite a multidisciplinary approach that includes broad-spectrum antibiotics, radical surgical debridement and hemodynamic support in an intensive care unit (ICU), mortality rates are still very high and can be as high as 88 % [15, 16]. Early diagnosis, aggressive resuscitation of the patient, administration of broad-spectrum antibiotics and aggressive radical surgical debridement(s), are the key of successful treatment [17]. A sudden increase in the number of patients with Fournier’s gangrene at Bugando Medical Centre (in northwestern Tanzania) in recent years and lack of published data on this subject prompted the authors to analyze this problem. This study was conducted in our setting to describe our experiences in the management of Fournier’s gangrene and to identify predictors of outcome among these patients.

## Methods

This was a descriptive retrospective study of patients with Fournier’s gangrene carried out at Bugando Medical Centre (BMC) in northwestern Tanzania between November 2006 and April 2014. Bugando Medical Centre is located in Mwanza city along the shore of Lake Victoria in the northwestern part of Tanzania. It is a tertiary care and teaching hospital for the Catholic University of Health and Allied Sciences-Bugando (CUHAS-Bugando) and other paramedics and has a bed capacity of 1000. BMC is one of the four largest referral hospitals in the country and serves as a referral centre for tertiary specialist care for a catchment population of approximately 13 million people from neighboring regions.

All patients who were diagnosed and managed for Fournier’s gangrene during the study period were included in the study. Patients who had incomplete data were excluded from the study. The diagnosis of Fournier’s gangrene was based on history and clinical examination. The details of patients were retrieved from the patient files kept in the medical record department, the surgical wards, operating theatre and laboratories. Data collected included age, gender, risk factors, etiology, clinical signs and symptoms, clinical parameters (heart rate, temperature, respiratory rate and blood pressure), laboratory findings (serum sodium, potassium, creatinine and bicarbonate, hematocrit and leukocyte count), duration of symptoms before admission and number of surgical debridement. The severity of Fournier’s gangrene on admission was assessed using the Fournier’s gangrene severity index (FGSI) score [12, 13]. We calculated FGSI from clinical (temperature, heart and respiratory rate) and laboratory parameters (serum sodium, potassium, creatinine and bicarbonate, haematocrit and leukocyte count) obtained on admission, as suggested by Laor et al. [18]. Each parameter is given 04 points, and FGSI is calculated by summing up the points of each parameter. The cutoff point is 9 so that when FGSI is >9, the probability of death is 75 % and when it is <9, the probability of survival is 78 %.

Patients were resuscitated where necessary with intravenous fluids, antibiotics (metronidazole, ceftriaxone and gentamicin), analgesics, and anti-tetanus prophylaxis, blood transfusions were also given. Investigations carried out included packed cell volume, urinalysis, blood sugar and chemistry, wound swab and biopsy for microbial culture, and HIV screening. Wound debridement was done for all patients (some more than once) under general or spinal anesthesia. The definitive management was done when the wound became clean with healthy granulation. The definitive management took the form of healing by secondary intention, by secondary closure, skin grafting, and skin flap rotation.

Mortality was defined as disease-related death during the hospital stay and the length of hospital stay was measured in days. The prognostic variables used in the outcome analysis were the patient’s age, gender, infection source, existing predisposing factors, the interval between the onset of symptoms and hospital admission, clinical findings, laboratory results, number of surgical debridement, whether or not colostomy was performed, occurrence of sepsis and FGSI.

### Definition of terms

*Systemic inflammatory response syndrome* was defined as the presence of two or more of the following, temperature >38 °C or <36 °C, tachycardia >90 beats/min, respiratory rate >20 breaths/min and white blood cell count >12 × 10^9^/L or <4 × 10^9^/L.

*Sepsis* was defined as bacterial infection of blood stream or body tissue.

*Leukocytosis* was defined as a white blood cell count of 10,000/mm^3^ or more.

*Anemia* was defined as hemoglobin levels <10 g/dl.

### Statistical data analysis

Statistical data analysis was performed using SPSS computer software version 17.0 (SPSS, Inc., Chicago, IL, USA). Data were summarized in the form of proportions and frequency tables for categorical variables. Continuous variables were summarized using median (+IQR) and ranges. The correlation of prognostic (independent) variables and outcome variables (mortality and length of hospital stay were studied by univariate analysis using Chi squared test and Fisher’s exact probability test. Statistically significant variables were entered into multivariate regression analysis using logistic regression. P values were reported as the result of two-tailed testing and P values less than 0.05 were considered as statistically significant.

### Ethical consideration

The study was performed according to the declaration of Helsinki and approved by the CUHAS-Bugando/BMC joint institutional ethic review committee before the commencement of the study. This research being a retrospective study where patient’s files kept in the medical record department, the surgical wards, operating theatre and laboratories have been used to gather the information, no written informed consent for participation in the study was obtained from participants or, where participants are children, a parent or guardian. The confidentiality of the patient was maintained at all time of the study and only file numbers were used instead of patient’s names.

## Results

**Fig. 1:**
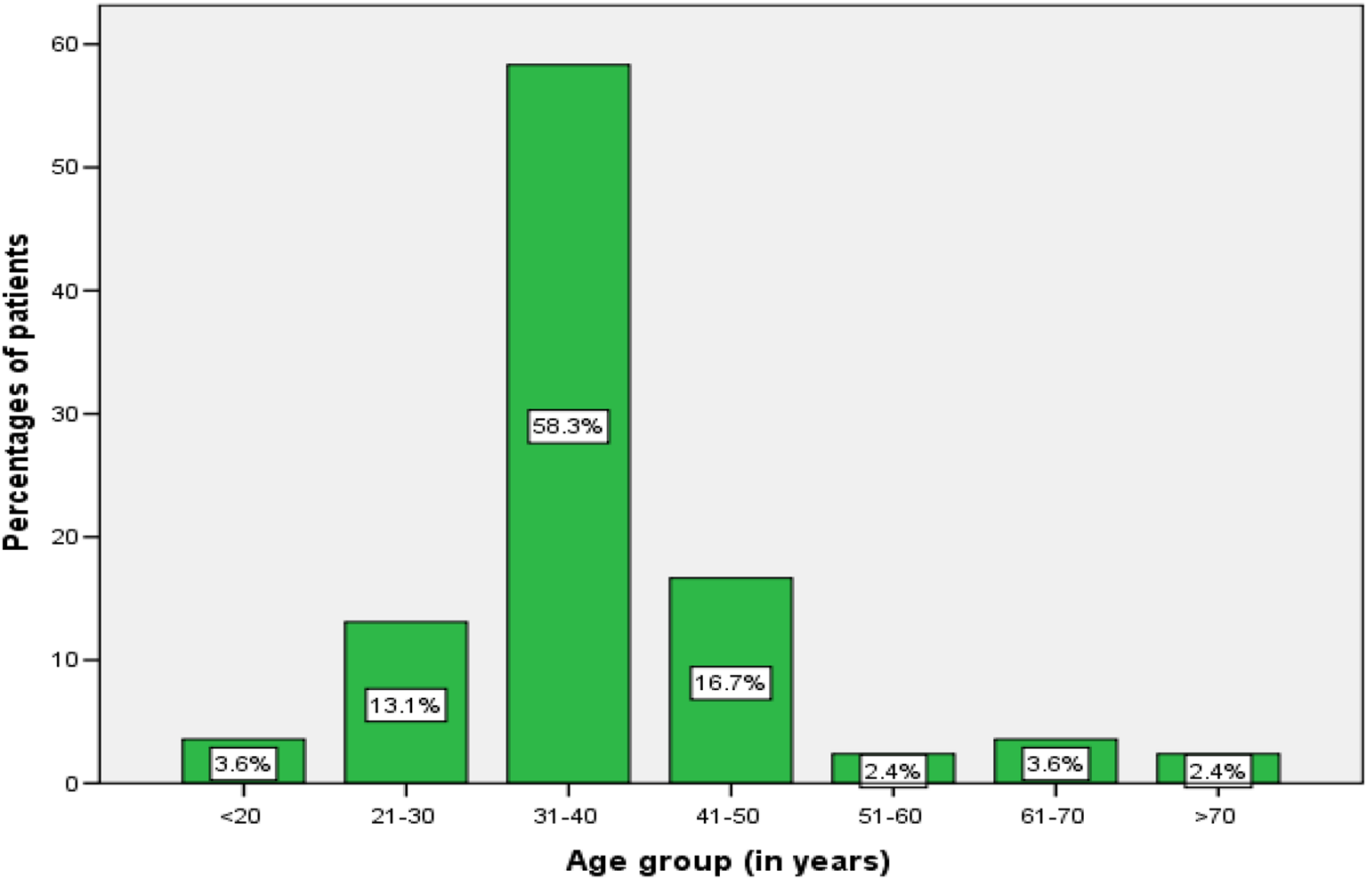
Distribution of patients according to the age group

A total of 88 patients were seen over a period of study with four patients excluded from the study on account of incomplete data, leaving 84 patients for the final analysis. There were 82 (97.6 %) males and 2 (2.4 %) females with a male to female ratio of 41: 1. The age of patients at presentation ranged from 15 to 76 years with a median of 34 years (+IQR of 32 to 41 years). The modal age group was 31–40 years accounting for 58.3 % of cases (Fig. 1). Most of patients, 68 (81.0 %) had either primary or no formal education and more than 80 % of them were unemployed. The majority of patients, 72 (85.7 %) came from the rural areas located a considerable distance from the study area and more than ninety percent of them had no identifiable health insurance.

Predisposing factors for Fournier’s gangrene were identified in 69 (82.1 %) patients of which diabetes mellitus was the leading predisposing factor associated with Fournier’s gangrene and accounted for 14 (16.7 %) patients. The predisposing factors for Fournier’s gangrene were not identified in 17.9 % of cases (Table 1).

**Table 1:** Distribution of patients according to predisposing/etiological factors

Predisposing/etiological factors	Number of patients	Percentages
Diabetes mellitus	14	16.7
Peri-anal abscess	11	13.1
HIV/AIDS	9	9.5
Post-inguinoscrotal herniorrhaphy	8	8.3
Perineal/scrotal trauma	8	8.3
Thrombosed haemorrhoids	7	8.3
Post-hydrocelectomy	5	6.0
Urethral stricture with watering cane perineum	2	2.4
Benign prostatic hyperplasia	2	2.4
Post-penile amputation	2	2.4
Urinary bladder injury with extravasation of urine	1	1.2
Predisposing factor not identified	15	17.9

The scrotum was the most frequent anatomical location involved accounting for 78.6 % of cases. Other anatomical location and extent are shown in Table 2.

**Table 2:** Anatomical location and extent of Fournier’s gangrene

Location	Frequency	Percentages
Scrotal	66	78.6
Perineal	4	4.8
Peri-anal	4	4.8
Thigh	3	3.6
Inguinal	2	2.4
Penis	2	2.4
Lumbo-sacral	1	1.2
Buttock	1	1.2
Lower anterior abdominal wall	1	1.2

The duration of symptoms before admission ranged from 1 to 18 days with a median of 4 days (+IQR of 2–8 days). The majority of patients, 64 (76.2 %) sought medical attention after more than 48 h of the onset of symptoms. The onset of symptoms was insidious in 48 (57.1 %) patients and abrupt in 36 (42.9 %) patients. Of the 84 patients, 62 (73.8 %) presented with established ulcers of the involved area, 12 (14.3 %) had established gangrene while the remaining 10 (11.9 %) presented with cellulitis, which later progressed to scrotal gangrene or resolved with treatment. All patients presented with fever, pain and discharge of sero-purulent material from the site. They also had swelling, redness, tenderness, and black dermal necrosis of the skin.

Hematological investigations revealed leukocytosis in all the patients (100 %), elevated ESR in 76 (90.5 %) and anemia (hemoglobin <10 g/dL) in 80 (95.2 %) patients. Out of 80 patients tested for HIV infection, nine (11.3 %) were HIV positive. Of the HIV positive patients, three (33.3 %) patients were known cases on ant-retroviral therapy (ARV) and the remaining 6 (66.7 %) patients were, CD 4+ count among HIV positive patients was available in only 5 patients and ranged from 113 to 678 cells/μl with a median of 236 cells/μl (+IQR of 232–240 cells/μl).

In this study, aerobic bacterial culture results were obtained in only 46 (54.8 %) patients. Of these, 38(82.6 %) had polymicrobial bacterial growth while 8 (17.4 %) had monomicrobial bacterial growth. *Escherichia coli* (28.3 %) and *Staphylococcus aureus* (17.4 %) were the most frequent bacterial organisms isolated (Table 3). Anaerobic cultures were not done due to lack of facility to perform this test. Antibacterial susceptibility testing revealed that most of pathogens isolates had multiple resistant to almost all tested antibiotics (such as ampicillin, augumentin, cotrimoxazole, tetracycline, penicillin, gentamicin, erythromycin, oxacillin etc.) except for Meropenem and imipenem, which were 100 % sensitive each respectively.

**Table 3:** Distribution of patients according to the type of micro-organisms isolated

Micro-organisms isolated	Frequency	Percentages
*Escherichia coli*	13	28.3
*Staphylococcus aureus*	8	17.4
*Klebsiella pneumonia*	7	15.2
ß-hemolytic streptococcus	6	13.0
*Acinetobacter spp*	4	8.7
*Pseudomonas aeruginosa*	2	4.3
*Proteus vulgaris*	2	4.3
Other rare spp	4	8.7

Before surgery, all patients underwent aggressive fluid resuscitation and were treated mostly with parenteral broad-spectrum triple antimicrobial agents, using a third-generation cephalosporin, an aminoglycoside and metronidazole and received hemodynamic support when required. Meropenem and imipenem, which were 100 % sensitive were given to only few patients who managed to purchase. Mechanical ventilation, continuous monitoring, and inotropic support were applied when necessary in patients with cardiopulmonary failure due to sepsis. All patients underwent radical surgical debridement, ranging from 1 to 8 procedures, with a median of 3 (+IQR of 1 to 5). Debridement consisted of excision of all necrotic tissue, cleansing with hydrogen peroxide, then saline and packed with dressings soaked in povidone-iodine. Orchidectomy was carried out unilaterally for gangrenous testes in 3 (3.6 %) patients. There were no adjunct surgeries such as suprapubic cystostomy, colostomy or penectomy. After the initial surgery, the wound was closely monitored, adequate nutrition was ensured to support wound healing and early enteral feeding was considered. The patients underwent repeated debridements as necessary and further necrotic tissues were debrided when needed under local or no anesthesia. Closure of wounds was commenced as soon as healthy, viable tissue allowed re-approximation. The majority of the patients, 65 (77.4 %) had wound closure by secondary closure. Other patients had their wound closed by skin grafting and flap rotation in 14 (16.7 %) and 5 (5.9 %) patients respectively.

The overall length of hospital stay (LOS) ranged from 8 to 56 days with a median of 28 days (+IQR of 26 to 32 days). The median LOS for non-survivors was 7 days (range 1–16 days). Factors predicting the length of hospital stay in univariate analysis were systemic inflammatory response syndrome on admission (OR = 2.4, 95 %CI (1.1–6.3), p = 0.034), late presentation >48 h (OR = 4.8, 95 % CI (3.2–6.9), p = 0.016), HIV positivity (OR = 5.2, 95 % CI (2.8–9.0), p = 0.003) and diabetes mellitus (OR = 2.1, 95 % CI (1.0–6.7), p = 0.011). According to multivariate logistic regression analysis, systemic inflammatory response syndrome [odds ratio (OR) = 3.9, 95 % confidence interval (CI): 1.5–8.5, p = 0.002) and diabetes mellitus (OR = 3.0, 95 % CI: 2.1–5.9, p = 0.044) were the main predictors of the length of hospital stay.

In this study, twenty-four patients died giving a mortality rate of 28.6 %. Univariate analysis revealed high mortality rates in the following conditions; advancing age >60 years (p = 0.003), hospital admission later than 48 h of onset of symptoms (p = 0.012), systemic inflammatory response syndrome on admission (p = 0.001), diabetes mellitus (p = 0.002), HIV positivity (p = 0.005), low CD 4 count (<200 μl/cells) and FGSI >9 (p = 0.003). According to multivariate logistic regression analysis; advancing age >60 years (Odds ratio = 2.4, 95 % CI (1.7–4.6), p = 0.002), late presentation >48 h (Odds ratio = 3.9, 95 % CI (2.2–9.4), p = 0.000), systemic inflammatory response syndrome on admission (Odds ratio = 7.3, 95 %CI (2.5–9.7), p = 0.016), diabetes mellitus (Odds ratio = 2.2, 95 % CI (1.7–5.9), p = 0.010), extension of infection to the abdominal wall (Odds ratio = 6.7, 95 %CI (3.8–9.0), low CD 4 count (<200 μl/cells) (Odds ratio = 4.9, 95 % (2.1–8.7), p = 0.021) and FGSI >9 (Odds ratio = 3.7, 95 % CI (2.8–5.6), p = 0.004).

## Discussion

Fournier’s gangrene is an acute, rapidly progressive and potentially fatal, infective necrotizing fasciitis affecting the external genitalia, perineal or perianal regions frequently due to a synergistic polymicrobial infection. It affects both sexes and all ages [1, 3–7]. In this study, the male predominance is in keeping with previous observations reported in studies done elsewhere [3–7, 15, 17, 18]. We could not find the reason for this gender distribution.

In this review, the median age of patients at presentation was 34 years, which is lower than the age reported in the literature [7, 16–18]. A study performed in Egypt by Ghnnam [19] reported the mean age of patients at diagnosis to be 51 years (21–72 years). Other studies reported older age at presentation [20–22]. We could not establish the reason for this age difference.

Fournier’s gangrene has been reported to be more prevalent in people with low socio-economic status [19]. This observation is reflected in our study where most of patients had either primary or no formal education and more than 75 % of them were unemployed. The majority of patients in the present study came from the rural areas located a considerable distance from the study area and more than 90 % of them had no identifiable health insurance. Similar observation was reported by others [19, 21, 22]. This observation has an implication on accessibility to health care facilities and awareness of the disease.

In keeping with other studies reported elsewhere [23–25], Diabetes mellitus was the most reported co-morbid disease associated with this pathology. Some authors estimate the prevalence of Diabetes mellitus among Fournier’s gangrene patients between 50 and 70 percent [23, 25]. Diabetes mellitus has been reported in literature as a risk factor for Fournier’s gangrene and associated with a more progressive and fatal outcome resulting from decreased phagocytic and intracellular bactericidal activity and neutrophil dysfunction [24],

In the present study, the scrotum was the most frequent anatomical site involved in more than three quarter percent of patients. Similar finding was also reported by Ayumba [26] in Kenya. Other studies reported anorectal region as the most common site involved [3, 7, 17, 19–21].

It has been reported that the interval between the onset of symptoms and initiation of treatment is a better predictor of outcome. In the present study most of patients presented late more than 48 h from the start of symptoms. This is in agreement with other studies in most developing countries [3, 17, 26]. Late presentation in our study may be attributed to lack of accessibility to health care facilities as more than eighty-five percent of patients came from the rural areas located a considerable distance from the study area and more than ninety percent of them had no identifiable health insurance.

The prevalence of HIV infection among patients with Fournier’s gangrene in the present study was 11.3 % that is significantly higher than 6.5 % [27] in the general population in Tanzania. The exact reason for the high prevalence of HIV infection in our patients is not known although it is possible that HIV positive patients have an increased risk of Fournier’s gangrene and are therefore overrepresented.

Various bacterial organisms both aerobes and anaerobes have been identified as agents that acts synergistically in causing the disease [5]. In our series, polymicrobial organisms were identified in more than eighty percent of cases. Most authorities believe the polymicrobial nature of Fournier gangrene is necessary to create the synergy of enzyme production that promotes rapid multiplication and spread of the infection [4, 7, 9]. The microbiological results of our study are similar to the literature, with *Escherichia coli* being the most prevalent organism [5, 9]. This centre has no facilities for anaerobic culture which probably accounted for the growth of only *Escherichia coli*. All the microorganisms isolated in this study showed multi-resistance to commonly used antibiotics except for Meropenem and imipenem which were all 100 % sensitive respectively. Unfortunately, these antibiotics are expensive for the level of economical development which subsists in this part of the developing world. The finding of polymicrobial infection and multiple resistant to commonly used antibiotics calls for immediately surgical intervention. Antibiotic susceptibility testing remains of paramount importance in the management of Fournier’s gangrene.

The main stays of treatment of Fournier’s gangrene are resuscitation, broad spectrum intravenous antibiotics and radical debridement [8, 28], same principles were followed in our centre. The role of hyperbaric oxygen and immuno-globulin has been well explored [29, 30] but it is not available in our centre as in most parts of the developing tropical African countries [29, 30] but instead all the patients had twice daily warm sitz bath after the initial debridement with a very good response.

The corporal bodies and testes are rarely affected because they have independent blood supplies that originate intra-abdominally. However, severe infections may penetrate the urogenital diaphragm and the perivesicle space and gain entry into the inguinal canal via the internal and external fascia of the spermatic cord [4, 28]. In this study, only three patients had orchidectomy as a result of gangrenous testis.

Some authors advocate suprapubic cystostomy and diverting colostomy to prevent wound contamination [28]. In the present study, there were no adjunct surgeries such as suprapubic cystostomy, diverting colostomy or penectomy. The two patients with urethral stricture with watering cane perineum identified in this study were managed by direct vision urethrotomy (DVU).

The length of hospital stay is an important measure of morbidity in which estimates of length of hospital stay are important for financial matters and accurate early estimates so as to facilitate better financial planning by the payers since it takes long for the Fournier’s gangrene to heal so increasing the costs as well as seen in other studies as well [14–16]. In this study, the overall median length of hospital stay was 28 days, a figure which is higher than that reported in other studies [16, 17, 28]. Prolonged length of hospital stay in our study was observed in patients with associated systemic inflammatory response syndrome and diabetes mellitus.

Early diagnosis, antimicrobial treatment with broad-spectrum combinations followed by prompt surgical debridement is the recommended management of Fournier’s gangrene [3, 7]. Even with this sequence of treatment approaches, mortality is high, 4–67 % [1, 19, 20, 23]. The high mortality reflects both the aggressive nature of the infection and the destructive effects of accompanying predisposing factors. In this study, the mortality rate was 28.6 % which is higher than that reported in other studies [10, 21, 28]. High mortality rate in the present study was attributed to advancing age (>60 years), late presentation (>48 h), systemic inflammatory response syndrome (SIRS), on admission, diabetes mellitus, extension of infection to the abdominal wall, Fournier’s gangrene severity score (FGSI) >9 and HIV infection with low CD4 count (<200 μl/cells). Addressing these factors responsible for the high mortality in our patients is mandatory to be able to reduce mortality associated with Fournier’s gangrene.

The major limitation of the study was poor documentation of medical findings and poor record keeping in view of the retrospective nature of the study. This might have introduced some bias in our findings. Microbiological analysis was only performed in less than one half of the patients. Therefore, the bacterial spectrum reported in this study might not representative. Failure to perform blood and anaerobic cultures were also a major limitation in this study. We however believe that despite of these limitations, some of the critical issues in Fournier’s gangrene have been well expounded by this study,

## Conclusion

Fournier’s gangrene remains a very severe disease in this part of Tanzania and is associated with a high mortality rate. Diabetes mellitus was the leading major illness associated with Fournier’s gangrene. The advanced age, late presentation, systemic inflammatory response syndrome on admission, diabetes mellitus, extension of infection to the abdominal wall, high Fournier’s gangrene severity score (>9) and HIV infection with low CD4 count (<200 μl/cells) were the main prognostic factors of mortality. Early recognition of infection associated with invasive and aggressive treatment is essential for attempting to reduce these prognostic factors. Antibiotic susceptibility testing remains of paramount importance in the management of this condition.

## References

[1] Corman JM, Moody JA, Aranson WL (1999). Fournier’s gangrene in a modern surgical setting: improved survival with aggressive management. Br J Urol Int.

[2] Fournier JA (1883). Gangrene foudroyante de la verge. Med Pract.

[3] Eke N (2000). Fournier’s gangrene: a review of 1726 cases. Br J Surg.

[4] Edino ST, Yakubu AA, Obidiaso A (2005). Fournier’s gangrene in a tertiary health facility in Nigeria. Afr J Urol.

[5] Yanar H, Taviloglu K, Ertekin C, Guloglu R, Zorba U, Cabioglu N, Baspinar I (2006). Fournier’s gangrene: risk factors and strategies for management. World J Surg.

[6] Korkut M, Içöz G, Dayangaç M, Akgün E, Yeniay L, Erdoğan O, Cal C (2003). Outcome analysis in patients with Fournier’s gangrene: report of 45 cases. Dis Colon Rectum.

[7] Villanueva-Saenz E, Martinez Hernandez-Magro P, Valdes Ovalle M, Montes Vega J, Alvarez-Tostado FJF (2002). Experience in management of Fournier’s gangrene. Tech Coloproctol.

[8] Paty R, Smith AD (1992). Gangrene and Fournier’s gangrene. Urol Clin North Am.

[9] Aghaji AE (2000). Fournier’s gangrene. Niger J Surg Sci.

[10] Jeong HJ, Park SC, Seo IY, Rim JS (2005). Prognostic factors in Fournier gangrene. Int J Urol.

[11] Yilmazlar T, Ozturk E, Alsoy A, Ozguc H (2007). Necrotizing soft tissue infections: APACHE II score, dissemination, and survival. World J Surg.

[12] Roghmann F, von Bodman C, Löppenberg B, Hinkel A, Palisaar J, Noldus J (2012). Is there a need for the Fournier’s gangrene severity index? Comparison of scoring systems for outcome prediction in patients with Fournier’s gangrene. BJU Int.

[13] Verma S, Sayana A, Kata S, Rai S (2012). Evaluatuion of the utility of the Fournier’s gangrene severity index in the Management of Fournier’s gangrene in North India: a multicentre retrospective Study. J Cutan Aesthet Surg.

[14] Sorensen MD, Krieger JN, Rivara FP, Klein MB, Wessells H (2009). Fournier’s gangrene: management and mortality predictors in a population based study. J Urol.

[15] Ugwumba FO, Nnabugwu II, Ozoemena OF (2012). Fournier’s Gangrene—analysis of management and outcome in South-Eastern Nigeria. S Afr J Surg.

[16] Sorensen MD, Krieger JN, Rivara FP, Broghammer JA, Klein MB, Mack CD (2009). Fournier’s gangrene: population based epidemiology and outcomes. J Urol.

[17] Benjelloun EB, Souiki T, Yakla N, Ousadden A, Mazaz K, Louchi A, Kanjaa N, Taleb KA (2013). Fournier’s gangrene: our experience with 50 patients and analysis of factors affecting mortality. World J Emerg Surg.

[18] Laor E, Palmer LS, Tolia BM, Reid RE, Winter HI (1995). Outcome prediction in patients with Fournier’s gangrene. J Urol.

[19] Ghnnam WM (2008). Fournier’s gangrene in Mansoura Egypt: A review of 74 cases. J Postgrad Med.

[20] Antonio A, Filho DC, Montovani LM (2010). Management of Fournier’s gangrene: experience of a University Hospital of Curitiba. J Braz Coll Surg.

[21] Khan I (2009). Experience in management of Fournier’s gangrene: a review of 19 Cases. Gomal J Med Sci.

[22] Malik AM, Sheikh S, Pathan R, Khan A, Sheikh U (2010). The spectrum of presentation and management of Fournier’s gangrene-an Experience of 73 Cases. J Pak Med Assoc.

[23] García A, Martín J, Vaquero A, Sánchez T, de Tomás J, Lago J, Turégano F (2011). Fournier’s gangrene: analysis of prognostic variables in 34 patients. Eur J Trauma Emerg Surg.

[24] Jarboui S, Jarraya H, Daldoul S, Zaouche A (2008). Étude clinique et thérapeutique et analyse pronostique des gangrènes du périnée. Presse Med.

[25] Dahm P, Roland FH, Vaslef SN, Moon RE, Price DT, Georgiade GS, Vieweg J (2000). Outcome analysis in patients with primary necrotizing fasciitis of the male genitalia. Urology.

[26] Ayumba BR, Magoha GA (1998). Epidemiological aspects of Fournier’s gangrene at Kenyatta National Hospital, Nairobi. East Afr Med J..

[27] Urassa M, Isingo R, Kumogola Y, Mwidunda P, Helelwa M, Changulucha J, Mngara J, Zaba B, Calleja T, Slaymaker E: Effect of PMTCT availability on choice of ANC in Mwanza and Magu districts and its impact on HIV sentinel surveillance in Tanzania. Report of ANC surveillance Mwanza and Magu Districts; 2007.

[28] Aji SA, Alhassan SU, Ujudud MM (2012). Fournier’s Gangrene: experience with management of 46 cases in a tertiary institution. Open J Urol.

[29] Korhonen K, Hirn M, Niinikoski J (1998). Hyperbaric oxygen in the treatment of Fournier’s gangrene. Eur J Surg.

[30] Wang C, Lau J: Hyperbaric oxygen therapy in treatment of hypoxic wounds. Hyperbaric Oxigen. 2001.

